# Usefulness of next-generation sequencing for laboratory diagnosis of rickettsiosis

**DOI:** 10.1371/journal.pntd.0012546

**Published:** 2024-10-09

**Authors:** Fanfan Xing, Chaowen Deng, Jinyue Huang, Yanfei Yuan, Zhendong Luo, Simon K. F. Lo, Susanna K. P. Lau, Patrick C. Y. Woo

**Affiliations:** 1 Department of Infectious Diseases and Microbiology, The University of Hong Kong ‐ Shenzhen Hospital, Shenzhen, Guangdong, China; 2 Department of Medical Imaging, The University of Hong Kong ‐ Shenzhen Hospital, Shenzhen, Guangdong, China; 3 Department of Microbiology, School of Clinical Medicine, Li Ka Shing Faculty of Medicine, The University of Hong Kong, Pokfulam, Hong Kong, China; 4 Doctoral Program in Translational Medicine and Department of Life Sciences, National Chung Hsing University, Taichung, Taiwan; 5 The iEGG and Animal Biotechnology Research Center, National Chung Hsing University, Taichung, Taiwan; Zhejiang University School of Medicine, CHINA

## Abstract

Rickettsiosis includes a diversity of culture-negative non-specific systemic infections. Laboratory diagnosis of rickettsiosis is often not easy. In this 12-month study, six patients with a variety of rickettsia infections of the spotted fever group, typhus group and scrub typhus were diagnosed directly or indirectly by metagenomic next-generation sequencing (mNGS). The patient with Japanese spotted fever was rapidly made when mNGS analysis of the patient’s blood revealed *Rickettsia japonica* sequences. For the two patients with *Rickettsia felis* chest infections, the bacterium was detected in the bronchoalveolar lavage of one case and lung biopsy of the other. Both patients had underlying malignancies, carcinoma of the breast and carcinoma of the lung respectively, and were on chemotherapy with immunosuppressive effect. For the remaining three patients who presented over a period of 13 weeks, all had fever, headache and the typical eschar. They also had increased serum transaminases and responded promptly to doxycycline. However, the Weil-Felix test results of all three patients were negative. Since we considered the three cases typical of rickettsiosis, we submitted their serum samples for mNGS analysis. Results showed that *Orientia tsutsugamushi* sequences were present in the serum of one case. In view of the positive mNGS results, we repeated the Weil-Felix test for the residual sera of all three patients and it revealed that those of the other two cases showed OX-19 titers of 1:640 and 1:160 respectively, inferring that these two patients probably had rickettsiosis of the typhus group. As for the patient positive for *O*. *tsutsugamushi* sequences, we also detected IgM for *O*. *tsutsugamushi* in the serum, which double confirmed that it was a case of scrub typhus. mNGS is an important molecular tool and can complement serology for laboratory diagnosis of rickettsiosis.

## Author summary

Rickettsiosis includes a diversity of systemic infections presented with non-specific symptoms. Laboratory diagnosis of rickettsiosis is often not easy. In this 12-month study, six patients with a variety of rickettsia infections were diagnosed directly or indirectly by metagenomic next-generation sequencing (mNGS). These include one patient with Japanese spotted fever in whom *Rickettsia japonica* sequences were detected in blood, two patients with *Rickettsia felis* chest infections in whom *R*. *felis* sequences were detected in respiratory samples, one patient with scrub typhus in whom *Orientia tsutsugamushi* sequences were detected in serum, and two patients with rickettsiosis of the typhus group. In particular, the last three patients had initial false-negative Weil-Felix test results performed in a central reference laboratory but the diagnosis of rickettsiosis was subsequently confirmed by mNGS and repeated serology. mNGS is an important diagnostic tool for rickettsiosis and other difficult-to-diagnose systemic infections.

## Introduction

The family Rickettsiaceae comprises two major pathogenic genera associated with human infections, namely *Rickettsia* and *Orientia* [1]. For the genus *Rickettsia*, it consists mainly of the spotted fever group (e.g. *R*. *conorii* [Mediterranean spotted fever], *R*. *felis*, *R*. *japonica*, *R*. *rickettsii* [Rocky Mountain spotted fever]) and the typhus group (e.g. *R*. *prowazekii* [epidemic typhus], *R*. *typhi* [murine typhus]); whereas for *Orientia*, there is only one major pathogenic species, *O*. *tsutsugamushi* [scrub typhus]. These Gram-negative intracellular bacteria are transmitted by arthropods, *R*. *conorii* and *R*. *rickettsii* by ticks, *R*. *prowazekii* by the human body louse, *R*. *typhi* by rat and cat fleas, and *O*. *tsutsugamushi* by chiggers. Clinically, rickettsiosis usually present with fever, headache and rash, which may be associated with inoculation eschar and/or localized lymphadenopathy [2]. In addition, neutropenia, thrombocytopenia and moderate increases in liver parenchymal enzymes are common. Since the disease often presents non-specifically and is sometimes mis-diagnosed, the global burden of rickettsiosis is difficult to estimate. However, seroepidemiology studies have shown that the infection is indeed not uncommon. For example, studies in Asia have shown that the general population has a median seroprevalence of 22% against *O*. *tsutsugamushi* [3–6].

Traditionally, laboratory diagnosis of rickettsiosis was mainly achieved by serology [2]. Although largely replaced by more specific antibody detection assays, the Weil-Felix test, based on cross-reactivity between a number of *Proteus* antigens and different members of the Rickettsiaceae family, continues to hold importance in resource-limited regions. Nevertheless, as the test is based on cross-reactivity, patients with Proteus infections may show false-positive results. Specific rickettsial antibody test, performed using the immunofluorescence technique, is currently the standard rickettsial antibody assay. However, these antibody tests are only available for the better characterized species associated with rickettsiosis, such as *R*. *rickettsii* and *R*. *typhi*, but not the newer species such as *R*. *felis* and *R*. *japonica*; and cross-reactions between the different species are also common. Polymerase chain reaction using serum samples collected during the early phase of the disease or eschar swabs could complement the serological tests [2], but it could be negative during the convalescent phase of the infection, particularly if the patient has received appropriate antibiotic treatment.

In the last few years, next-generation sequencing (NGS) has emerged as a technology for laboratory diagnosis of many culture-negative infections [7,8]. We have recently reported its application in confirming the first case of listeria meningitis in a patient with autoantibody against interferon gamma and a patient with fatal *Nocardia kroppenstedtii* bacteremic pneumonia and empyema thoracis as well as understanding the spectrum of Q fever, fungal infections, Whipple’s disease and culture-negative meningitis and encephalitis [8–13]. In this study, we report the usefulness of NGS for the laboratory diagnosis of the relatively less common rickettsiosis as well as re-confirming the diagnosis of scrub typhus and other rickettsia infections with false-negative Weil-Felix test results.

## Materials and methods

### Ethical statement

The study was approved by the Medical Ethics Committee of The University of Hong Kong ‐ Shenzhen Hospital, with patient consent waived because this is a retrospective study and no additional testing was performed ([2022]120).

### Patients

This was a retrospective study conducted over a 12-month period (1^st^ December 2021 to 30^st^ November 2022) in The University of Hong Kong ‐ Shenzhen Hospital. This 1,700-bed multi-specialty hospital was established in 2012 and provides primary to tertiary medical services to the residents of Shenzhen city and as a medical supplementation of Hong Kong residents in both inpatient and outpatient settings. Shenzhen is the first Special Economic Zone with an estimated population of nearly 18 million people including a large migrant population from other regions in China in a variety of jobs. Shenzhen is located in the southeast of Guangdong Province, which is an endemic area of scrub typhus and immediately north to Hong Kong. The clinical details, laboratory data and radiological findings of all patients with rickettsiosis were retrieved from the hospital electronic record system and analyzed. The diagnosis of rickettsiosis was made based on a combination of clinical manifestation, imaging, serological or molecular test results.

### Microbiological methods

Clinical specimens were collected and handled according to standard protocols [14]. The BacT/ALERT 3D(240) blood culture system (Becton Dickinson, Maryland, USA) was used. Respiratory specimens, including bronchoalveolar lavage fluid (BAL), tracheal aspirate and sputum were each inoculated onto blood agar plate, chocolate agar plate, MacConkey agar plate, Sabouraud dextrose agar (SDA) plate and triphenyltetrazolium chloride SDA plate. All suspected isolates were identified based on morphological characteristics, conventional biochemical methods and matrix-assisted laser desorption/ionization-time of flight mass spectrometry (MALDI-TOF MS) Microflex LT/SH (Bruker Daltonics, Bremen, Germany) and the spectra analyzed with IVD MALDI Biotyper 2.3 and reference library DB-9607 (Bruker Daltonics). Direct detection of *Pneumocystis jirovecii* and acid-fast bacilli (AFB) in respiratory samples were performed by Grocott-Gomori methenamine silver (GMS) stain and Ziehl-Neelsen stain, respectively. Respiratory samples of the patients were sent to KingMed Diagnostic (Guangzhou, China) for mycobacterial culture. 1,3-β-ᴅ-glucan detection was performed using Test Kit for the Detection of Fungus 1,3-β-ᴅ-Glucan (Photometric Assay) (A & C Biological Ltd, Zhanjiang, China). According to the manufactures’ protocols, real-time PCR for *Mycobacterium tuberculosis* and *Mycoplasma pneumoniae* were performed using *M*. *tuberculosis* DNA Fluorescence Diagnostic Kit and *M*. *pneumoniae* DNA Fluorescence Diagnostic Kit (PCR-Fluorescence Probing) (Sansure Biotech, Hunan, China), respectively; and real-time RT-PCR for severe acute respiratory syndrome coronavirus 2 (SARS-CoV-2) was performed using 2019-nCoV Nucleic Acid Test Kit (Biogerm, Shanghai, China).

### Weil-Felix test

Serum for Weil-Felix test was sent to Shenzhen Center for Disease Control and Prevention (SZCDC) routinely, whenever rickettsiosis was suspected. The repeated Weil-Felix test was performed by KingMed Diagnostic (Guangzhou, China) using Febrile Antigens (Rapid Labs Ltd, United Kingdom) and the tube agglutination test method.

### Orientia tsutsugamushi IgM antibody test

*O*. *tsutsugamushi* IgM antibody was detected using the OneStep Scrub Typhus IgM Serum/WB/Plasma RapiDip InstaTest (Diagnostic Automation/Cortez Diagnostics, California, USA). The test was performed and results interpreted according to manufacturer’s instructions. Briefly, 10 μL whole blood was added to the test strip and the strip was placed vertically in a microtiter well. Then, 90–120 μL of the Chase Buffer solution was added into the well and the results were interpreted in 15 minutes. The test result was considered positive when a control line and a test line appeared in the test area and negative if only the control line appeared. A positive result indicated that the Cortez Scrub Typhus IgM rapid dipstick detected antibodies to mixture of *O*. *tsutsugamushi* derived recombinant antigens.

### Next-generation sequencing

Ethylene Diamine Tetraacetic Acid (EDTA)-treated blood, tissues, and BAL samples were collected from the patients and sent to KingMed Diagnostic (Guangzhou, China) for metagenomic NGS (mNGS) analysis of pathogenic microorganisms [15]. Briefly, DNA was extracted from the clinical specimens using TIANamp DNA Kit (TIANGEN, Beijing, China). The extracted DNA was treated with the enzyme mixture in the KS619-DNAmN24 kit and the fragments analysed using an automatic nucleic acid protein analyser Qseq-100 (Bioptic, Jiangsu Province, China). The fragments used for library preparation were within the range of 250–350 bp. Sequencing was performed using the Illumina NextSeq 550Dx platform and data acquisition conducted through single-end sequencing with a read-length of 75 bp. The sequencing data generated by mNGS was processed by bcl2fastq software (v2.20.0.422) to demultiplex sequencing data and convert base calling files into raw fastq-format files. The adapters, low-quality sequences and duplicated sequences were removed using Fastp (v0.23.1). Sequence reads originated from human were filtered by aligning the resulting data to human reference (hg38) using BWA (0.7.17-r1188, Burrow-Wheeler Aligner). Subsequently, the sequencing reads were compared to classification reference genomes database which contained 13,214 bacteria, 9,811 viruses, 3,180 fungi, and 405 parasites which were downloaded from NCBI (ftp://ftp.ncbi.nlm.nih.gov/genomes/). Self-developed software was used to calculate the number of reads per 20 million (RP20M) for species or genus level and visualization of sequence alignment. The Jurkat cell model, *Curtobacterium citreum*, *Schizosaccharomyces pombe* and plasmid of MS2 bacteriophage were used as positive controls.

## Results

### *Rickettsia japonica* infection

*Case 1*. A 60-year-old Chinese woman with good past health was admitted because of fever, chills and headache for six days. One day after the onset of fever, she also developed skin rash on the trunk. She was prescribed with oral cefuroxime but did not respond to the treatment. On admission, her body temperature was 40°C. Macular rash was observed on the four limbs and the trunk. Parenchymal liver enzymes were elevated (Table 1). Serum procalcitonin level was 1.99 ng/mL (normal range < 0.25 ng/mL). Blood culture was performed and empirical intravenous ceftriaxone and oral doxycycline were commenced. mNGS analysis of the blood sample showed sequence reads of *Rickettsia japonica* (n = 6) (Table 2), representing around 0.05% of its genome. The fever responded to the ceftriaxone and doxycycline and the rash subsided gradually. Blood culture was negative. Weil-Felix test performed by the SZCDC showed OX-2, OX-19 and OX-K titers of < 1:20 (Table 3).

**Table 1:** Results of blood tests for patients in the present study.

Blood tests	Results					
	Case 1	Case 2	Case 3	Case 4	Case 5	Case 6
**WBC (×10** ^**9**^ **/L)**	8.16	7.55	2.79	13.41	9.69	3.13
**Hemoglobin (g/L)**	134	118	94	110	152	138
**Platelet (×10** ^**9**^ **/L)**	119	453	25	357	164	153
**Neutrophil (×10** ^**9**^ **/L)**	7.24	5.88	2.27	8.49	6.91	2.58
**Lymphocyte (×10** ^**9**^ **/L)**	0.61	1.18	0.35	3.55	2.32	0.43
**Monocyte (×10** ^**9**^ **/L)**	0.3	0.32	0.17	0.99	0.4	0.12
**Eosinophil (×10** ^**9**^ **/L)**	0	0.15	0	0.03	0	0
**ALT (U/L)**	42.7	28.6	59.7	123.7	218.5	378.4
**AST (U/L)**	61.6	32.7	22.9	165.7	143.4	699.8
**ALP (U/L)**	104	106	88	83	82	201
**TBIL (μmol/L)**	23	9.7	6.4	7.2	7	13.4
**GGT (U/L)**	60.5	36.2	37.8	33	125.5	66.9
**Creatinine (μmol/L)**	78	60	40	66	61	61
**D-dimmer (μg/mL)**	5.03	Not done	Not done	Not done	Not done	11.02
**PT (s)**	14.2	13.4	12.9	13.7	14.2	13.7
**aPTT (s)**	43.1	40.5	41.6	47	36.7	38.9
**Fibrinogen (g/L)**	3.82	5.53	4.95	4.67	4.29	2.73
**CRP (mg/L)**	173.75	38.81	103.41	52.37	36.55	39.82
**PCT (ng/mL)**	1.99	0.1	0.125	0.4	0.75	0.37

Abbreviation: ALT, Alanine aminotransferase; AST, Aspartate aminotransferase; ALP, alkaline phosphatase; TBIL, total bilirubin; GGT, γ-glutamyl transferase; PT, prothrombin time; aPTT, activated partial thromboplastin time; CRP, C-reactive protein; PCT, procalcitonin.

**Table 2:** Results of mNGS analysis for patients in the present study.

mNGS analysis	Results					
	Case 1	Case 2	Case 3	Case 4	Case 5	Case 6
**Specimen**	EDTA blood	BAL	Lung nodule	Serum	Serum	Serum
**Organism detected (number of sequence reads)**	*Rickettsia japonica* (6), Torque teno midi virus 2 (5)	*Rickettsia felis* (1469), *Streptococcus mitis* (15748), *Streptococcus pneumoniae* (3818), *Klebsiella pneumoniae* (45), HHV-7 (9), *Prevotella melaninogenica* (14316), *Neisseria mucosa* (4968), *Actinomyces oris* (4136), *Lautropia mirabilis* (9897), *Veillonella atypica* (2178), *Schaalia odontolytica* (4574), *Porphyromonas somerae* (2085), *Haemophilus parainfluenzae* (2324), *Gemella haemolysans* (2067), *Treponema medium* (999), *Rothia mucilaginosa* (1557), *Granulicatella adiacens* (1202), *Peptostreptococcus stomatis* (1182), *Parvimonas micra* (955)	*R*. *felis* (12), *Aspergillus fumigatus* (185), CMV (87), HHV-6 (11), Torque teno virus (38), *Leclercia adecarboxylata* (8), *Ralstonia mannitolilytica* (7), *Bifidobacterium breve* (4), *Candida parapsilosis* (2), EBV (1)	None	*O*. *tsutsugamushi* (2)	*Staphylococcus epidermidis* (4), *Acinetobacter baumannii* (4)

Abbreviation: mNGS, metagenomic next-generation sequencing; EDTA, Ethylene diamine tetraacetic acid; BAL, bronchoalveolar lavage fluid; HHV, human herpes virus; CMV, cytomegalovirus; EBV, Epstein-Barr virus.

**Table 3:** Results of rickettsia serology tests for patients in the present study.

Rickettsia serology tests	Results					
	Case 1	Case 2	Case 3	Case 4	Case 5	Case 6
***Admission Weil-Felix test***						
**OX-19**	< 1:20	Not done	Not done	< 1:20	< 1:20	< 1:20
**OX-2**	< 1:20	Not done	Not done	< 1:20	< 1:20	< 1:20
**OX-K**	< 1:20	Not done	Not done	< 1:20	< 1:20	< 1:20
***Repeated Weil-Felix test***						
**OX-19**	Not done	Not done	Not done	1:640	< 1:20	1:160
**OX-2**	Not done	Not done	Not done	< 1:20	< 1:20	< 1:20
**OX-K**	Not done	Not done	Not done	< 1:20	< 1:20	< 1:20
***Specific rickettsia serology test***						
***Orientia tsutsugamushi* IgM**	Not done	Not done	Not done	Negative	Positive	Negative

### *Rickettsia felis* infection

**Fig 1:**
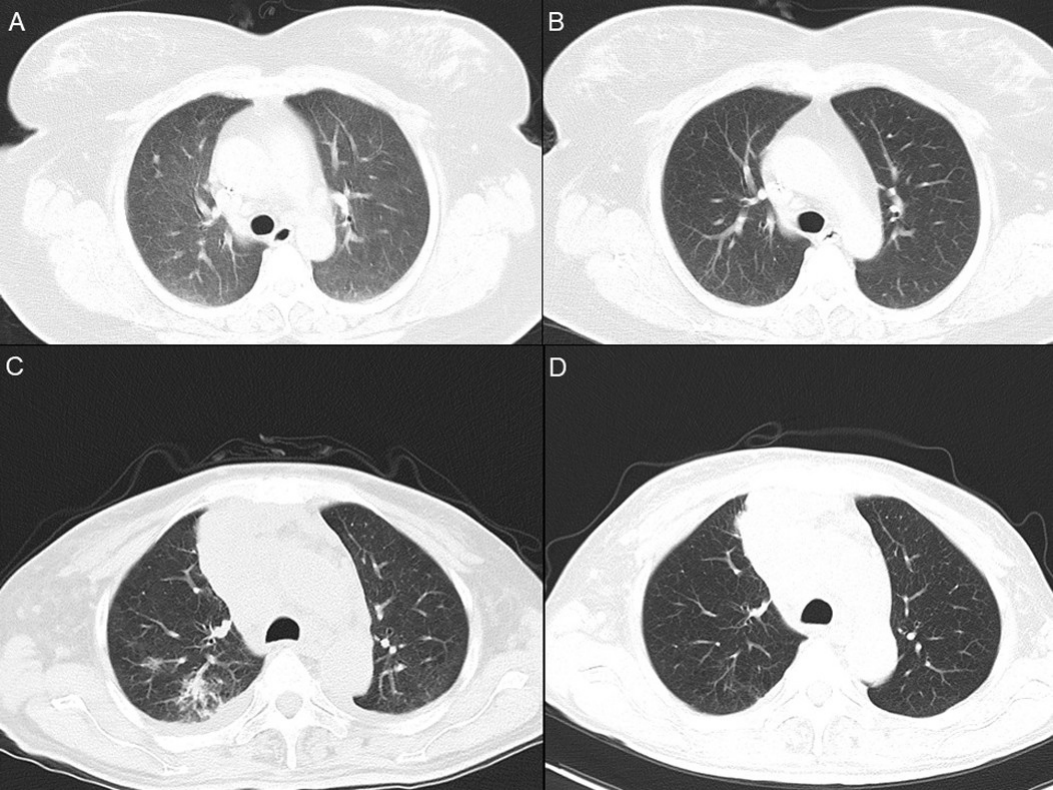
Thoracic computed tomography scan of Case 2 and 3 before and after specific antibiotic treatment. Panel A: Case 2, day 9 after admission, showing diffuse interstitial infiltrates and ground-glass nodules in both lungs; Panel B: Case 2, day 40 after admission, showing clearance of infiltrates and nodules; Panel C: Case 3, day 5 after admission, showing infiltrates in the posterior segment of the upper lobe of the right lung; Panel D: Case 3, day 64 after admission, showing clearance infiltrates.

*Case 2*. A 48-year-old Chinese woman was admitted because of fever and shortness of breath for one day. The patient was diagnosed to have carcinoma of the breast five months ago and was treated with radical mastectomy followed by 10 courses of cyclophosphamide, doxorubicin and paclitaxel. On admission, her body temperature was 38°C. Her SaO_2_ was 92% on room air. Blood results are shown in Table 1. Computed tomography (CT) scan of the thorax revealed interstitial infiltrates and sporadic solid nodules in both lungs (Fig 1A). Blood culture was performed and bronchoalveolar lavage was collected for microbiological investigations. Empirical intravenous cefoperazone-sulbactam was commenced. *Klebsiella pneumoniae*, viridans streptococci and *Neisseria* species were isolated from the BAL. mNGS analysis of the BAL showed sequence reads of *Rickettsia felis* (n = 1,469, representing around 0.05% of its genome), *Streptococcus pneumoniae* (n = 3,818), *K*. *pneumoniae* (n = 45), and other microbes (Table 2). Oral doxycycline was added for the treatment of possible *R*. *felis* infection. The fever and shortness of breath started to improve three days after the commencement of doxycycline. Direct GMS staining for *Pneumocystis jirovecii* and fungal and mycobacterial cultures were negative. CT scan of the thorax performed 6 weeks after admission showed disappearance of the interstitial infiltrates in the lungs (Fig 1B).

*Case 3*. A 56-year-old Chinese man was admitted because of cough and shortness of breath for two days. The patient was diagnosed to have carcinoma of the lung two months ago and was treated with paclitaxel, cisplatin, bevacizumab and crizotinib. Two days before this admission, he developed cough, shortness of breath, oral pain and facial and neck swelling. On admission, his body temperature was 38°C. His SaO_2_ was 92.3% on room air. Blood results are shown in Table 1. CT scan of the thorax revealed a mass in the right upper mediastinum; infiltrates, consolidation and multiple nodules in both lungs; multiple cervical, supraclavicular and mediastinal lymph nodes; and pleural effusion; compatible with the underlying malignant condition and a possible recently developed infection (Fig 1C). Blood culture was performed and sputum was collected for microbiological investigations. Empirical intravenous amoxicillin-clavulanate was commenced. Despite antibiotic treatment, the fever persisted. Sputum culture recovered *Candida albicans*. Serum 1,3-β-ᴅ-glucan was positive. Eight days after admission, amoxicillin-clavulanate was stopped and intravenous piperacillin-tazobactam and fluconazole were commenced. Twelve days after admission, biopsy of a recently appeared subpleural nodule in the left lung was performed. Histopathological studies showed chronic inflammatory cells infiltration, granuloma-like structures with multinucleated giant cells, and hyphae-like structures. Fever gradually subsided. mNGS analysis of the tissue sample showed sequence reads of *Aspergillus fumigatus* (n = 185), *Rickettsia felis* (n = 12, representing <0.01% of its genome), and other microbes (Table 2). Piperacillin-tazobactam and fluconazole were stopped and intravenous voriconazole and oral doxycycline were commenced for the treatment of possible aspergillosis and *R*. *felis* infection. The patient remained afebrile and was discharged. Thoracic CT scan repeated 9 weeks later showed that both the pulmonary infiltrates and pleural effusion have been largely resolved (Fig 1D).

### *Orientia tsutsugamushi* and other rickettsia infection

**Fig 2:**
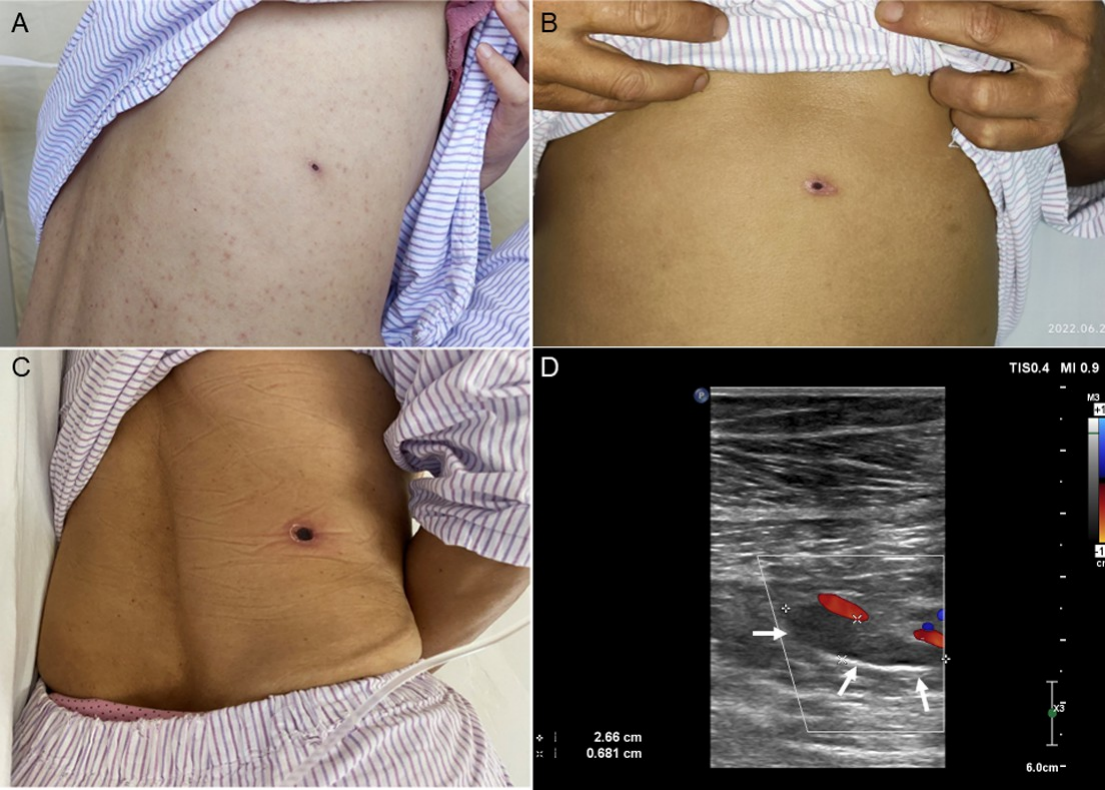
Clinical-radiological assessment of Case 4, 5 and 6. Panel A: eschar on lower back of Case 4; Panel B: eschar on abdominal wall of Case 5; Panel C: eschar on lower back of Case 6; Panel D: color doppler flow imaging for the left leg of Case 6, showing a 2.66 cm × 0.681 cm hypoechoic clot in the intermuscular vein lumen of the left leg (arrow), non-compressibility of the vein, and no spontaneous venous flow in the local intermuscular veins.

*Case 4*. A 48-year-old Chinese woman with good past health was admitted because of fever and headache for 12 days. The fever and headache were associated with fatigue and poor appetite. Eight days before admission, skin rash developed on both thighs and spread to the trunk and both upper limbs. She was prescribed with oral cephalosporin and antihistamines by another hospital but did not respond to the treatment. On admission to our hospital, her body temperature was 37.9°C. Purpuric rash was observed on the four limbs and the trunk. An eschar was noted on the right lateral chest wall (Fig 2A). Multiple painless cervical lymph nodes were palpable. Parenchymal liver enzymes were elevated (Table 1). A clinical diagnosis of rickettsia infection was made and oral doxycycline was commenced. Although the fever responded promptly and the rash subsided gradually, Weil-Felix test performed by the SZCDC showed OX-2, OX-19 and OX-K titers of <1:20 (Table 3).

*Case 5*. A 42-year-old Chinese male sanitation worker with good past health was admitted because of fever and headache for one week. The fever and headache were associated with poor appetite. He was self-medicated with oral cephalosporin but he did not respond to the treatment. On admission, his body temperature was 39.9°C. An eschar was noted at the center of the abdominal wall (Fig 2B). Parenchymal liver enzymes were elevated (Table 1). A clinical diagnosis of rickettsia infection was made and oral doxycycline was commenced. Although the fever and headache responded promptly, Weil-Felix test performed by the SZCDC showed OX-2, OX-19 and OX-K titers of < 1:20 (Table 3).

*Case 6*. A 58-year-old Chinese female sanitation worker with good past health was admitted because of fever and headache for one week. The fever and headache were associated with nausea and vomiting. Six days before admission, she was prescribed with oral oseltamivir but she did not respond to the treatment. On admission, her body temperature was 38.6°C. An eschar was noted on the right lower back (Fig 2C). She had lymphopenia and thrombocytopenia and the liver enzymes were markedly elevated (Table 1). The serum D-dimer level was 11.02 μg/mL (normal < 0.5 μg/mL), although the prothrombin time and activated partial thromboplastin time were in normal range. A clinical diagnosis of rickettsia infection was made and oral doxycycline was commenced. Doppler ultrasound examination revealed deep vein thrombosis of the lower limbs (Fig 2D). Low molecular weight heparin and then rivaroxaban were prescribed. Although the fever, headache and deep vein thrombosis responded promptly, Weil-Felix test performed by the SZCDC showed OX-2, OX-19 and OX-K titers of < 1:20 (Table 3).

*NGS and retrospective confirmation of Case 4*, *5 and 6 as O*. *tsutsugamushi and other rickettsia infection*. Since all three patients were highly suspected to have rickettsia infections despite negative Weil-Felix test, their serum samples were sent for mNGS analysis. Results revealed that sequence reads of *O*. *tsutsugamushi* (n = 2, representing <0.01% of its genome) were present in the serum sample of Case 5, but not Case 4 or 6 (Table 2). Due to the positive NGS results, Weil-Felix test was repeated on the residual serum samples of Case 4, 5 and 6 that were previously tested “negative” by SZCDC, and *O*. *tsutsugamushi* IgM detection was also performed. Results showed OX-19 titers of 1:640 and 1:160 (OX-2 and OX-K titers < 1:20) for Case 4 and 6 respectively and positive *O*. *tsutsugamushi* IgM for Case 5 (Table 3).

## Discussion

In this study, we demonstrated the usefulness of NGS for the laboratory diagnosis for a variety of rickettsia infections, including the relatively uncommon rickettsiosis such as *R*. *japonica* and *R*. *felis* infections. *R*. *japonica*, associated with severe tick-borne rickettsiosis of the spotted fever group, was first reported in Japan in 1984 [16]. Japanese spotted fever is mainly reported from Japan, with some cases in South Korea, the Philippines and Thailand [17]. However, Japanese spotted fever is not common in China. It has been reported recently that Japanese spotted fever could be severe with disseminated intravascular coagulation, and sometimes it can even be fatal or associated with serious sequalae such as purpura fulminans requiring amputation or skin graft [18]. In the present study, our patient with Japanese spotted fever (Case 1) presented non-specifically with fever, chills, headache and a macular rash but without an eschar. The diagnosis was rapidly made when mNGS analysis revealed sequence reads of *R*. *japonica* and the patient responded promptly to doxycycline without any complications. As for *R*. *felis*, it is mainly transmitted by the cat flea (*Ctenocephalides felis*), although it has also been detected in a variety of arthropods, such as mosquitoes, ticks and mites [19]. Similar to the other rickettsiosis, flea-borne spotted fever is usually a systemic disease. *R*. *felis* has never been reported to be detected in respiratory samples. In the present report, *R*. *felis* was detected in the BAL of Case 2 and lung biopsy tissue of Case 3. Both patients had underlying malignancies, carcinoma of the breast (Case 2) and carcinoma of the lung (Case 3) and were on chemotherapy with immunosuppressive effect. In addition to *R*. *felis*, other potential pathogens were also detected in the patients’ BAL samples, *Streptococcus pneumoniae* and *K*. *pneumoniae* in Case 2 and *Aspergillus fumigatus* in Case 3. Although *R*. *felis* may not be the only cause of the chest infections in these two patients, treatment with doxycycline as well as antibiotics/antifungals resulted in clearance of the pulmonary infiltrates as demonstrated in the interval CT scan of the thorax.

In addition to picking up patients with *R*. *felis* and *R*. *japonica* infections of the spotted fever group, NGS is also useful for rectifying the diagnosis of scrub typhus and other rickettsia infections with false-negative Weil-Felix test results. In this study, Case 4, Case 5 and Case 6 presented to our hospital over a period of 13 weeks. All three patients had fever, headache and the typical eschar which was almost considered as diagnostic for rickettsia infection. In addition, they all had increased serum transaminase levels and they responded promptly to doxycycline treatment. However, the Weil-Felix test results of all three patients performed by the SZCDC were negative. Since we considered the three cases typical of rickettsiosis but with negative Weil-Felix test results, we submitted the serum samples of them for mNGS analysis. Results showed that *O*. *tsutsugamushi* sequences were indeed present in the serum of Case 5, although not for Case 4 and 6. In view of the positive NGS results for Case 5, we repeated the Weil-Felix test for the residual serum samples of all three patients and it showed that the serum samples of Case 4 and Case 6 showed OX-19 titers of 1:640 and 1:160 respectively, with OX-2 and OX-K titers < 1:20. In general, *Proteus* OX-19 antigen reacts strongly with sera of patients with rickettsiosis of either the typhus group or the spotted fever group, *Proteus* OX-2 antigen reacts strongly with sera of patients with rickettsiosis of the spotted fever group, and *Proteus* OX-K antigen reacts strongly with sera of patients with scrub typhus. Since the sera of Case 4 and 6 have high OX-19 titers but OX-2 and OX-K titers of < 1:20, it was likely that these two patients had rickettsiosis of the typhus group. As for Case 5, in addition to the presence of *O*. *tsutsugamushi* sequence reads, we also detected IgM for *O*. *tsutsugamushi* in the serum, which double confirmed that Case 5 was a case of scrub typhus. It is of note that deep vein thrombosis is extremely rare in patient with rickettsiosis, with only a few cases reported in the literature [20–25]. Although NGS is a useful diagnostic tool, it is also important to point out that NGS is not a laboratory test with perfect sensitivity and specificity. The imperfect sensitivity was well-illustrated by Case 4 and 6 in the present scenario, where NGS results were negative for the two cases of scrub typhus; whereas its low specificity was demonstrated by both the present and our previous studies, where NGS analysis often resulted in detecting sequence reads of a few dozens of microbes, which could be the genuine pathogens, colonizers or contaminants [8,10,12,13]. For example, in Case 2 of the present study, apart from *R*. *felis*, sequence reads of 18 additional microbes were also detected by NGS, of which all were considered as colonizers or contaminants (Table 2). In clinical practice, NGS and serology should complement each other for laboratory diagnosis of rickettsiosis, and all NGS results must be interpreted in the clinical context of the patient. Afterall, NGS is of particular importance for early diagnosis and treatment of rickettsiosis, and has a potential role in guiding public health interventions when an outbreak may have occurred.

NGS would continue to be an important diagnostic tool for culture-negative systemic infections. For culture-negative systemic infections, such as Q fever, Whipple disease and rickettsiosis, failure to make a diagnosis is mainly due to the difficulty for the clinician to recognize the disease or lack of laboratory support to confirm the diagnosis. For Q fever, in modern cities where farms are not commonly found, clinicians are often unfamiliar with the diverse presentations of this disease. Moreover, the infection is often self-limited or it may be treated empirically with doxycycline. For Whipple disease, manifestations can be diverse and it can present in different forms, including transient and acute infection, asymptomatic carriage, localized Whipple disease such as culture-negative endocarditis, classic and systemic Whipple disease and Whipple disease in association with immunosuppression. Failure of entertaining the infection as a differential diagnosis will result in failing of ordering the corresponding laboratory tests. In addition to the difficulty in recognizing the possible diagnosis clinically, laboratory diagnosis of these infections is also challenging. Isolating the corresponding bacteria in these infections is notoriously difficult and usually not attempted. Serology, if available, could be associated with false-negative results; as for example, in Cases 4, 5 and 6 in the present study. This is partly due to the low incidence of the diseases and hence the technicians may be unfamiliar with the protocol and interpretation of the test results. Although specific PCR tests have improved the sensitivity of picking up the microbes, it still requires the clinician to have a high index of suspicion so that the corresponding PCR test is requested. As NGS is a one-technology-for-all-pathogen laboratory test, it can be performed even if the clinician does not have a good idea of what the culprit of the infection is. Moreover, it can also save the trouble of designing and maintaining multiple sets of PCR reactions for these difficult cases. Our recent studies on Q fever and Whipple disease [11,12], and the present study on rickettsiosis, have illustrated the usefulness of NGS for the diagnosis of the corresponding infections. In particular, all the cases with sequence reads of *Tropheryma whipplei* detected in the respiratory samples of the patients were atypical of Whipple disease [12]; and the present study is also the first one to pick up *R*. *felis* in respiratory samples. Further systematic studies should be carried out to document the usefulness, cost-effectiveness as well as clinical and public health impact of NGS on culture-negative infections. Clinical guidelines should also be formulated on the role of NGS in various clinical settings.

## References

[1] Gillespie JJ, Salje J. *Orientia* and *Rickettsia*: different flowers from the same garden. Curr Opin Microbiol. 2023 Aug;74:102318. doi: 10.1016/j.mib.2023.102318 Epub 2023 Apr 18. . 37080115

[2] Blanton LS. The Rickettsioses: A Practical Update. Infect Dis Clin North Am. 2019 Mar;33(1):213–229. doi: 10.1016/j.idc.2018.10.010 ; PMCID: PMC6364315. 30712763 PMC6364315

[3] Bonell A, Lubell Y, Newton PN, Crump JA, Paris DH. Estimating the burden of scrub typhus: A systematic review. PLoS Negl Trop Dis. 2017 Sep 25;11(9):e0005838. doi: 10.1371/journal.pntd.0005838 ; PMCID: PMC5634655. 28945755 PMC5634655

[4] Mayxay M, Castonguay-Vanier J, Chansamouth V, Dubot-Pérès A, Paris DH, Phetsouvanh R, Tangkhabuanbutra J, Douangdala P, Inthalath S, Souvannasing P, Slesak G, Tongyoo N, Chanthongthip A, Panyanouvong P, Sibounheuang B, Phommasone K, Dohnt M, Phonekeo D, Hongvanthong B, Xayadeth S, Ketmayoon P, Blacksell SD, Moore CE, Craig SB, Burns MA, von Sonnenburg F, Corwin A, de Lamballerie X, González IJ, Christophel EM, Cawthorne A, Bell D, Newton PN. Causes of non-malarial fever in Laos: a prospective study. Lancet Glob Health. 2013 Jul;1(1):e46–54. doi: 10.1016/S2214-109X(13)70008-1 ; PMCID: PMC3986032. 24748368 PMC3986032

[5] Taylor AJ, Paris DH, Newton PN. A Systematic Review of Mortality from Untreated Scrub Typhus (*Orientia tsutsugamushi*). PLoS Negl Trop Dis. 2015 Aug 14;9(8):e0003971. doi: 10.1371/journal.pntd.0003971 ; PMCID: PMC4537241. 26274584 PMC4537241

[6] McGready R, Ashley EA, Wuthiekanun V, Tan SO, Pimanpanarak M, Viladpai-Nguen SJ, Jesadapanpong W, Blacksell SD, Peacock SJ, Paris DH, Day NP, Singhasivanon P, White NJ, Nosten F. Arthropod borne disease: the leading cause of fever in pregnancy on the Thai-Burmese border. PLoS Negl Trop Dis. 2010 Nov 16;4(11):e888. doi: 10.1371/journal.pntd.0000888 ; PMCID: PMC2982829. 21103369 PMC2982829

[7] Wilson MR, Naccache SN, Samayoa E, Biagtan M, Bashir H, Yu G, et al. Actionable diagnosis of neuroleptospirosis by next-generation sequencing. N Engl J Med. 2014 Jun 19;370(25):2408–17. doi: 10.1056/NEJMoa1401268 Epub 2014 Jun 4. ; PMCID: PMC4134948. 24896819 PMC4134948

[8] Tsang CC, Teng JLL, Lau SKP, Woo PCY. Rapid Genomic Diagnosis of Fungal Infections in the Age of Next-Generation Sequencing. J Fungi (Basel). 2021 Aug 5;7(8):636. doi: 10.3390/jof7080636 ; PMCID: PMC8398552. 34436175 PMC8398552

[9] Xing F, Hung DLL, Lo SKF, Chen S, Lau SKP, Woo PCY. Next-generation sequencing-based diagnosis of bacteremic *Listeria monocytogenes* meningitis in a patient with anti-interferon gamma autoantibodies: a case report. Infect Microb Dis 2022;4(1):44–46. doi: 10.1097/IM9.0000000000000080

[10] Xing F, Xia Y, Lu Q, Lo SKF, Lau SKP, Woo PCY. Rapid diagnosis of fatal *Nocardia kroppenstedtii* bacteremic pneumonia and empyema thoracis by next-generation sequencing: a case report. Front Med (Lausanne). 2023 Jul 18;10:1226126. doi: 10.3389/fmed.2023.1226126 ; PMCID: PMC10392123. 37534314 PMC10392123

[11] Xing F, Ye H, Deng C, Sun L, Yuan Y, Lu Q, et al. Diverse and atypical manifestations of Q fever in a metropolitan city hospital: Emerging role of next-generation sequencing for laboratory diagnosis of *Coxiella burnetii*. PLoS Negl Trop Dis. 2022 Apr 20;16(4):e0010364. doi: 10.1371/journal.pntd.0010364 ; PMCID: PMC9060374. 35442979 PMC9060374

[12] Xing F, Lo SW, Liu M, Deng C, Ye H, Sun L, et al. Emergence of *Tropheryma whipplei* detection in respiratory samples by next-generation sequencing: Pathogen or innocent bystander? J Infect. 2023 Feb;86(2):154–225. doi: 10.1016/j.jinf.2022.12.004 Epub 2022 Dec 9. . 36509359

[13] Xing F, Yang Q, Deng C, Sun L, Luo Z, Ye H, et al. Clinical impact of next-generation sequencing on laboratory diagnosis of suspected culture-negative meningitis and encephalitis. J Infect. 2022 Nov;85(5):573–607. doi: 10.1016/j.jinf.2022.08.026 Epub 2022 Aug 28. . 36037832

[14] Carroll KC, Pfaller MA. 2019. Manual of clinical microbiology, 12th ed. ASM Press, Washington, DC.

[15] Wanghu H, Li Y, Huang J, Pu K, Guo F, Zhong P, Wang T, Yuan J, Yu Y, Chen J, Liu J, Chen JJ, Hu C. A novel synthetic nucleic acid mixture for quantification of microbes by mNGS. Microb Genom. 2024 Feb;10(2):001199. doi: 10.1099/mgen.0.001199 ; PMCID: PMC10926700. 38358316 PMC10926700

[16] Mahara F. Three Weil-Felix reaction (OX2) positive cases with skin eruptions and high fever. Journal of Anan Medical Association 1984;68:4–7

[17] Satjanadumrong J, Robinson MT, Hughes T, Blacksell SD. Distribution and Ecological Drivers of Spotted Fever Group Rickettsia in Asia. Ecohealth. 2019 Dec;16(4):611–626. doi: 10.1007/s10393-019-01409-3 Epub 2019 Apr 15. ; PMCID: PMC6910891. 30993545 PMC6910891

[18] Gao S, Li L, Zhou X, Dai X, Lu L, Chen Y, et al. Fatal *Rickettsia japonica* Infection Complicating Disseminated Intravascular Coagulation in Yichang, China. Infect Drug Resist. 2022 Nov 11;15:6613–6623. doi: 10.2147/IDR.S383917 ; PMCID: PMC9664911. 36386421 PMC9664911

[19] Blanton LS, Walker DH. Flea-Borne Rickettsioses and Rickettsiae. Am J Trop Med Hyg. 2017 Jan 11;96(1):53–56. doi: 10.4269/ajtmh.16-0537 Epub 2016 Oct 31. ; PMCID: PMC5239709. 27799640 PMC5239709

[20] Del Prete E, Pizzanelli C, Moretti P, Cosottini M, Bonuccelli U. Mediterranean spotted fever: an unusual clinical and neuroradiological presentation. Neurol Sci. 2015 Nov;36(11):2141–3. doi: 10.1007/s10072-015-2313-z Epub 2015 Jul 8. . 26152799

[21] Vicente V, Alberca I, Ruiz R, Herrero I, Gonzalez R, Portugal J. Coagulation abnormalities in patients with Mediterranean spotted fever. J Infect Dis. 1986 Jan;153(1):128–31. doi: 10.1093/infdis/153.1.128 . 3941278

[22] Nagaki Y, Hayasaka S, Kadoi C, Matsumoto M, Sakagami T. Branch retinal vein occlusion in the right eye and retinal hemorrhage in the left in a patient with classical Tsutsugamushi disease. Jpn J Ophthalmol. 2001 Jan-Feb;45(1):108–10. doi: 10.1016/s0021-5155(00)00293-8 . 11163055

[23] Alió J, Ruiz-Beltran R, Herrero-Herrero JI, Hernandez E, Guinaldo V, Millan A. Retinal manifestations of Mediterranean spotted fever. Ophthalmologica. 1987;195(1):31–7. doi: 10.1159/000309777 . 3658334

[24] Potasman I, Bassan HM. Pulmonary embolism complicating murine typhus. J R Soc Med. 1986 Jun;79(6):367–8. doi: 10.1177/014107688607900617 ; PMCID: PMC1290351. 3723539 PMC1290351

[25] Aten AH. Thrombophlebitis retina in abdominal typhus. Ophthalmologica. 1949 Jun;117(6):366. . 18132912

